# Accelerating AOP Development in the AOP-Wiki with AI: A Practical Road Map for the Community

**DOI:** 10.1021/acs.est.6c05148

**Published:** 2026-05-18

**Authors:** You Song, Vikas Kumar, Shihori Tanabe, Daniel L. Villeneuve, Clemens Wittwehr

**Affiliations:** † Norwegian Institute for Water Research (NIVA), Sognsveien 72, 0855 Oslo, Norway; ‡ Pere Virgili Health Research Institute (IISPV), C/Dr. Mallafrè Guasch 4, Edifici Modular, Hospital Universitari de Tarragona Joan XXIII, 43007 Tarragona, Spain; § German Federal Institute for Risk Assessment (BfR), Max-Dohrn-Strasse 8-10, 10589 Berlin, Germany; ∥ National Institute of Health Sciences, Center for Biological Safety and Research, Division of Risk Assessment, 3-25-26, Tonomachi, Kawasaki-ku, Kawasaki 210-9501, Japan; ⊥ Office of Chemical Safety and Pollution Prevention, Risk Assessment Support Division, U.S. Environmental Protection Agency (US EPA), 6201 Congdon Boulevard, Duluth, Minnesota 55804-2595, United States; # 99013 European Commission, Joint Research Centre (JRC), Via Enrico Fermi, 2749, 21027 Ispra, VA, Italy

**Keywords:** adverse outcome pathway (AOP), AOP-Wiki, artificial intelligence (AI), large language model (LLM), human−AI hybrid workflow, evidence synthesis

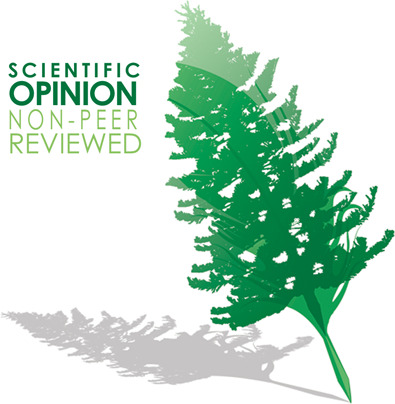

## Graphical abstract

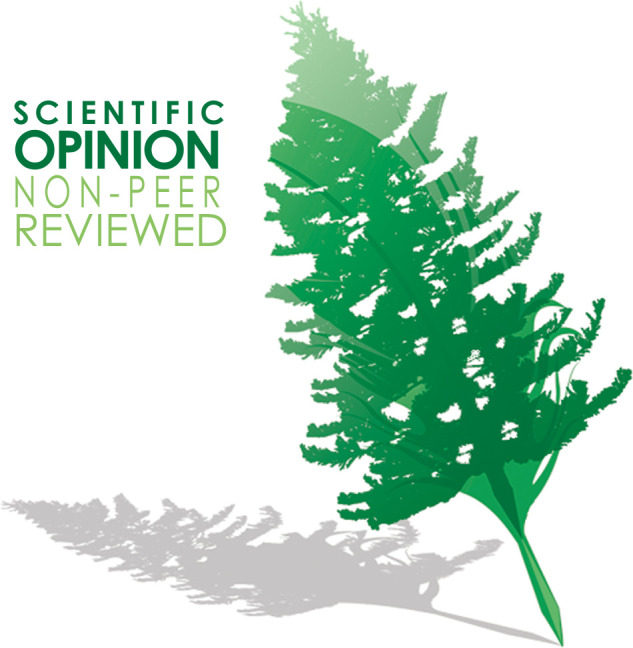

## Bottlenecks in AOP Development

Over the past 15 years, the adverse outcome pathway (AOP) framework has become a foundational element of next-generation risk assessment (NGRA), providing a structured, mechanistic representation of toxicological causality, from molecular initiating events (MIE), through key events (KE), to adverse outcomes (AO) of regulatory relevance.^1^ The framework serves two primary purposes: to organize existing knowledge and identify critical gaps and to provide mechanistic justification for the use of bioactivity data generated by new approach methodologies (NAMs) in regulatory decision-making.

Although the number of entries in the AOP-Wiki (https://aopwiki.org/) continues to increase, progress has been incremental, and the proportion of fully curated, high-quality, regulatory-endorsed AOPs remains low. Only a small fraction of the potential landscape has reached a level suitable for regulatory consideration (42 endorsed vs 561 total; accessed January 21, 2026). This imbalance reflects the reality that AOP development, according to AOP-Wiki and OECD guidance,^2^ remains largely manual, time-intensive, and cognitively demanding. Developers must draw transdisciplinary expertise; apply consistent, ontology-aligned terminology; critically appraise diverse literature; integrate heterogeneous data; justify causal linkages; and evaluate the strength of evidence using international standards.^2^ While this labor-intensive workflow limits scalability and slows the maturation of the AOP Knowledge Base, AI-based tools may help streamline discrete tasks without replacing the need for expert judgment.

These limitations extend beyond individual entries. As computational, predictive, and meta-analytic approaches increasingly rely on AOP-Wiki content, the scarcity of high-quality AOPs has downstream implications, including reduced model robustness and the risk of misleading inferences. Similarly, the limited availability of reviewed and endorsed AOPs remains a significant bottleneck for wider regulatory uptake.

## AI-Accelerated AOP Development

Artificial intelligence (AI) has the potential to accelerate AOP development by supporting time-consuming tasks and improving consistency. Text-mining tools can assist with rapid screening of scientific literature to identify mechanistic evidence and candidate KEs or KE relationships (KERs). However, AOP development requires understanding causal biological sequences, rather than identifying co-occurring terms, and many existing natural-language processing tools were not designed for AOP-specific applications. Additional AI approaches, including structured scientific statements, knowledge-graph construction, ontology support, and causal inference, offer complementary capabilities relevant to different stages of AOP development, even if not yet widely applied to AOPs (Table 1).

**Table 1:** Representative AI Tools That Are Relevant for AOP Development

stage in AOP development	functional role	AI tool (programming skill needed? √ = yes; × = no)	link (reference)
preassessment of biological plausibility	causal inference; reasoning; quick validation	DoWhy (√); ChatGPT (×); Claude (×); Gemini (×); DeepSeek (×)	py-why/dowhy; chat.openai.com; claude.ai; gemini.google.com; deepseek.com
ontology mapping and KE harmonization	ontology mapping; terminology alignment; consistency checking	OntoGPT (√); BioPortal APIs (√); DeepOnto (√)	monarch/ontogpt; bioontology.org; KRR-Oxford/DeepOnto
knowledge mining	literature mining; causal knowledge discovery	AOP-helpFinder (×); SciBERT (√); PubMedBERT (√); CauseNet (×)	aop-helpfinder.u-paris-sciences.fr; allenai/scibert; huggingface; causenet.org
evidence extraction	mechanistic evidence extraction; causal statement extraction	AOP-Bot (√); S2CIE (√); spaCy (√); SciSpacy (√)	Crispae/AOPbotvisualizer; insilicohub; spacy.io; allenai/scispacy
weight of evidence assessment (OECD AOP Handbook)	evidence integration; retrieval-augmented generation (RAG)-based validation; reliable QA	ChatAOP (×); AOP-Smart (√); ChatGPT (×); Claude (×); Gemini (×); DeepSeek (×)	SKIG report; qinjiang-lab/AOP-Smart; chat.openai.com; claude.ai; gemini.google.com; deepseek.com
compliance self-check (coach checklist)	reporting; summarizing; guideline compliance	ChatGPT (×); Claude (×); Gemini (×)	chat.openai.com; claude.ai; gemini.google.com

A common barrier for many “everyday” AOP developers is that advanced AI tools often require programming skills or complex workflow integration; user-friendly solutions are therefore critical. Widely accessible large language model (LLM)-based systems already integrate smoothly into routine scientific work and can enhance AOP development without specialized expertise. Recent evaluations show that LLMs can identify MIEs, map KEs, and summarize causal linkages directly from the literature, reducing manual effort and improving evidence organization.^3^ For tasks such as ontology alignment, harmonization with existing AOP-Wiki content, and draft text preparation, LLMs generate reproducible outputs that promote consistency. Rather than replacing expert judgment, these models function as assistive tools, allowing developers to focus on interpretation, integration, and validation.

AI is most effective within clearly defined boundaries. Focused objectives, such as summarizing mechanistic findings, harmonizing terminology, or drafting initial KE/KER descriptions from curated evidence, yield reliable results. In contrast, broad exploratory searches, loosely defined inference, or evaluation of conflicting findings remains error-prone and requires expert oversight. A recognized limitation of LLMs is their tendency to generate plausible but incorrect information (“hallucinations”), for example, suggesting unsupported KERs, underscoring the need for careful expert validation. Until fully automated pipelines become feasible, a hybrid human–AI workflow remains the most reliable approach. AI accelerates labor-intensive steps, while experts ensure biological plausibility, contextual relevance, and scientific rigor. This balance enables faster AOP development without compromising reliability.

AI is not a substitute for transdisciplinary expert judgment, but a catalyst for more efficient and standardized AOP creation. When integrated into a hybrid workflow that includes various quality check points and guided by structured prompts grounded in established evaluation criteria (Figure 1), AI enables more rapid development while maintaining scientific integrity. Prompts aligned with guidance such as the OECD AOP Handbook,^2^ the AOP Coaching Checklist,^4^ or related frameworks, requesting biological plausibility, essentiality, empirical support, and ontology-aligned terminology, help keep AI outputs within scope and improve reliability.

**Figure 1:**
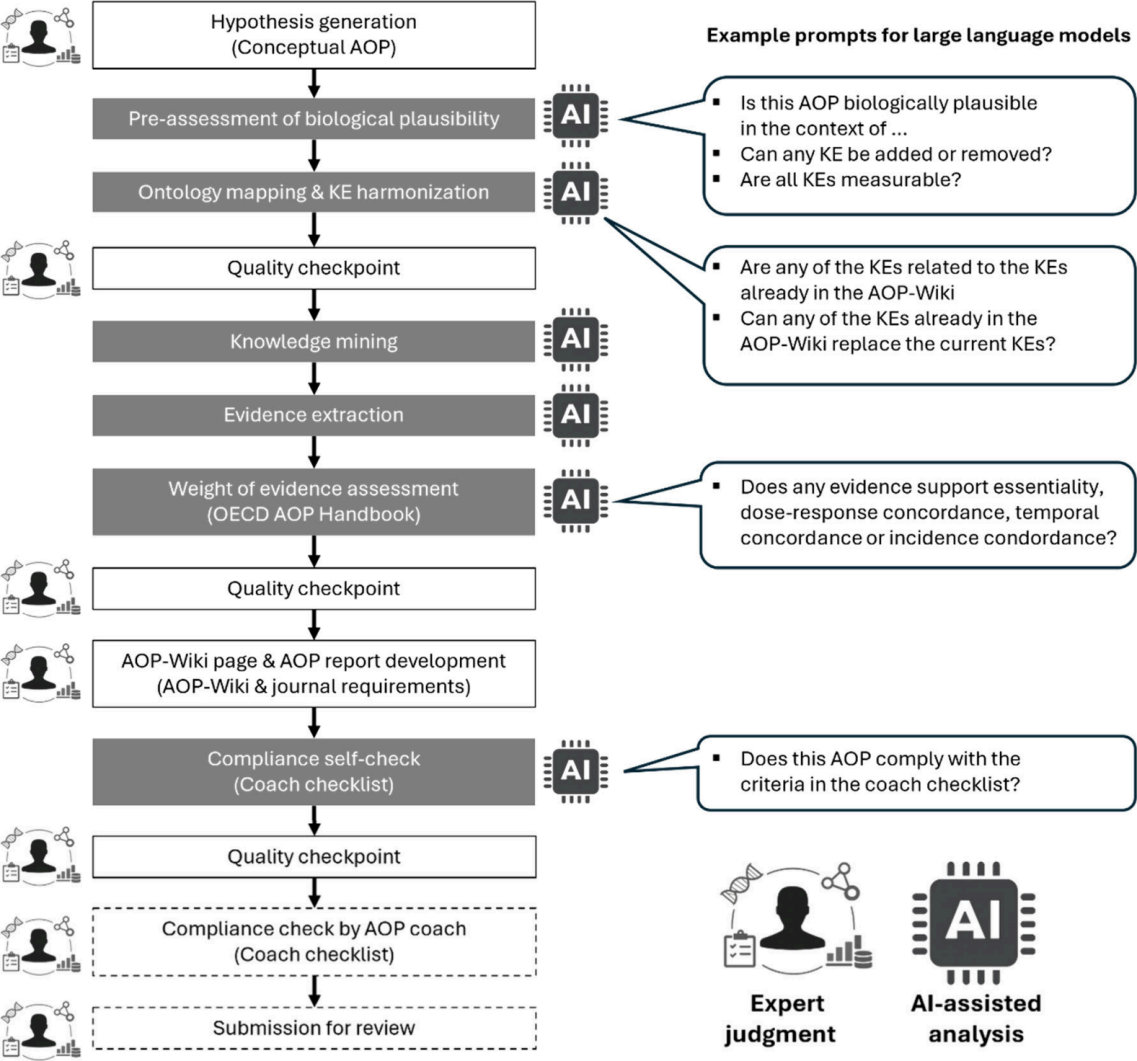
Proposed human–AI hybrid workflow for accelerating AOP development.

## The Path Forward: A Community Road Map

Realizing the potential of AI to accelerate AOP development requires coordinated, community-wide action. This calls for a balanced road map that combines near-term, low-effort “quick wins” with longer-term structural developments. The road map delineates near-term efforts (0–2 years) to enable AI-assisted AOP drafting, midterm actions (2–5 years) to integrate and scale semiautomated workflows and shared infrastructure, and long-term ambitions (≥5 years) to achieve network-level intelligence, automation, and continuously updated AOP knowledge systems. In this context, near-term efforts include AI-assisted literature triage, terminology harmonization, and semiautomated extraction of causal statements; midterm actions involve integration of these tools into shared workflows and infrastructure; and long-term ambitions encompass network-level automation and continuously updated AOP knowledge systems. The priority is not simply adopting new tools, but integrating them into transparent workflows that complement expert judgment.

The European Commission Joint Research Centre (JRC) is spearheading AI-enabled AOP development through the AI4AOP initiative, piloting practical workflows such as ontology-to-KE mapping (in collaboration with The University of North Carolina at Chapel Hill), contributing to the OECD’s Omics2AOP project, and leveraging community input to test and refine AI-generated KERs.^5^ These activities provide immediately usable outputs while informing future AI integration in the AOP-Wiki and align with community-driven plans for AOP-Wiki 3.0, which will modernize data structures, improve interoperability, and embed AI-assisted functions.^5^

To support these advances, AI-assisted workflows must be open, reproducible, and aligned with AOP-Wiki standards. Short-term opportunities include AI-assisted literature triage, automated terminology harmonization for new KEs and KERs, and semiautomated extraction of causal statements, steps that already perform reliably and require minimal curator training. Progress will also depend on shared training data sets and harmonized ontologies to address persistent terminology inconsistencies identified in recent AOP-Wiki evaluations.^5^

Building capacity across the community is essential, as many contributors lack computational expertise and access to experienced AOP developers or coaches, which remains a key bottleneck for broader participation. Practical workflow templates, lightweight training modules, and shared prompts aligned with OECD evaluation criteria can help lower these entry barriers. Community involvement is also critical. Initiatives that draw on broad contributor input and “crowd wisdom” demonstrate that individual developers can generate robust mechanistic insights when supported by structured, AI-assisted workflows. Embedding AI-enabled features, such as automated literature suggestions, ontology recommendations, or enhanced search tools like ChatAOP (a retrieval-augmented generation example applied to AOP-Wiki content),^5^ can further reduce reliance on intensive expert coaching and enhance accessibility. With transparent governance, shared evaluation standards, and appropriate expert oversight, AI can deliver immediate efficiency gains while enabling a scalable, evidence-driven pathway for future AOP-Wiki development.
